# Prediction of ovarian cancer prognosis and response to chemotherapy by a serum-based multiparametric biomarker panel

**DOI:** 10.1038/sj.bjc.6604630

**Published:** 2008-09-02

**Authors:** K Oikonomopoulou, L Li, Y Zheng, I Simon, R L Wolfert, D Valik, M Nekulova, M Simickova, T Frgala, E P Diamandis

**Affiliations:** 1 Department of Laboratory Medicine and Pathobiology, University of Toronto, Toronto, ON M5G1L5, Canada; 2 Department of Pathology and Laboratory Medicine, Mount Sinai Hospital, Toronto, ON M5G1X5, Canada; 3 Fred Hutchinson Cancer Research Center, Public Health Sciences Division, Seattle, WA 98109, USA; 4 diaDexus Inc., 343 Oyster Point Blvd., South San Francisco, CA 94080, USA; 5 Department of Laboratory Medicine, Masaryk Memorial Cancer Institute, Zluty kopec 7, Brno 656 53, Czech Republic

**Keywords:** human tissue kallikreins, serine proteases, ovarian cancer, biomarkers, multiparametric analysis, prediction of therapy

## Abstract

Currently, there are no effective biomarkers for ovarian cancer prognosis or prediction of therapeutic response. The objective of this study was to examine a panel of 10 serum biochemical parameters for their ability to predict response to chemotherapy, progression and survival of ovarian cancer patients. Sera from ovarian cancer patients were collected prior and during chemotherapy and were analysed by enzyme-linked immunosorbent assay for CA125, kallikreins 5, 6, 7, 8, 10 and 11, B7-H4, regenerating protein IV and Spondin-2. The odds ratio and hazard ratio and their 95% confidence interval (95% CI) were calculated. Time-dependent receiver-operating characteristic (ROC) curves were utilised to evaluate the prognostic performance of the biomarkers. The levels of several markers at baseline (c_0_), or after the first chemotherapy cycle (rc_1_), predicted chemotherapy response and overall or progression-free survival in univariate analysis. A multiparametric model (c_0_ of CA125, KLK5, KLK7 and rc_1_ of CA125) provided predictive accuracy with area under the ROC curve (AUC) of 0.82 (0.62 after correction for overfitting). Another marker combination (c_0_ of KLK7, KLK10, B7-H4, Spondin-2) was useful in predicting short-term (1-year) survival with an AUC of 0.89 (0.74 after correction for overfitting). All markers examined, except KLK7 and regenerating protein IV, were powerful predictors of time to progression (TTP) among chemotherapy responders. Individual and panels of biomarkers from the kallikrein family (and other families) can predict response to chemotherapy, overall survival, short-term (1-year) survival, progression-free survival and TTP of ovarian cancer patients treated with chemotherapy.

Ovarian cancer is the most lethal gynaecologic malignancy, ranking fifth in mortality among all carcinomas in women (American Cancer Society, 2007; Jemal *et al*, 2007). The high mortality rate has not improved substantially over the past years, despite our better understanding of the molecular events underlying malignancy and the availability of improved surgical techniques and novel chemotherapeutic agents (Marsden *et al*, 2000; American Cancer Society, 2007). The major reason for the poor prognosis of ovarian cancer is that over 75% of patients have disseminated disease (International Federation of Gynaecology and Obstetrics (FIGO) stage III or IV) at diagnosis (Marsden *et al*, 2000). These patients have a 5-year survival rate less than 30%, whereas patients with localised disease at diagnosis have an over 80% survival rate within 5 years (Nguyen *et al*, 1993; Bast, 2003).

One screening method for ovarian cancer is based on serum levels of CA125, a tumour-associated glycoprotein of unknown function. Nowadays, measurement of serum CA125 is considered essential for monitoring response to treatment (Van der Berg *et al*, 1988; Tuxen *et al*, 1995; Rustin *et al*, 1996) but has a limited value for general population screening (Tuxen *et al*, 1995; Zanotti and Kennedy, 1999). Furthermore, the clinical value of routinely measuring CA125 during follow-up after initial treatment is uncertain due to its limitations. One important limitation of serum CA125 is that its levels are not elevated in 50% of women with stage I disease and up to 30% of patients with more advanced disease (Tuxen *et al*, 1995). Its value as a screening test is even more uncertain, due to its poor specificity. Serum CA125 is elevated in a proportion of patients with diverse malignancies, such as colon, breast, endometrial, lung, liver and pancreatic tumours (Tuxen *et al*, 1995). Its levels can also be elevated during menstruation or pregnancy, as well as in other benign gynaecological conditions, such as endometriosis, peritonitis or cirrhosis, especially with ascites production (Tuxen *et al*, 1995, Meyer and Rustin, 2000).

Novel methods and markers with improved sensitivity and specificity are needed for early detection of ovarian cancer (Mills *et al*, 2001; Bast, 2003). High-throughput technologies, such as gene microarrays and proteomic approaches (Rai *et al*, 2002; Adib *et al*, 2004; Hibbs *et al*, 2004; Lu *et al*, 2004; Santin *et al*, 2004; Zhang *et al*, 2004), despite their limitations thus far (Diamandis, 2004; van der Merwe *et al*, 2007) have identified several molecular markers of early disease. Some promising novel ovarian cancer biomarkers include mesothelin (Huang *et al*, 2006) and human epididymis protein 4 (HE4) (Drapkin *et al*, 2005). In addition, among the novel markers are the human tissue kallikreins (kallikrein proteins (KLKs); reviewed in Borgono *et al* (2004) and Borgono and Diamandis (2004)).

Kallikreins are a family of 15 secreted serine proteases encoded by conserved genes, which are tandemly localised on chromosome 19q13.4 (Borgono *et al*, 2004). The kallikrein proteins are widely expressed in different tissues, during physiological or pathological conditions (Borgono *et al*, 2004; Shaw and Diamandis, 2007). Furthermore, several kallikreins are coexpressed in tissues or fluids (Borgono and Diamandis, 2004), suggesting their involvement in proteolytic cascades (Borgono and Diamandis, 2004; Pampalakis and Sotiropoulou, 2007; Yoon *et al*, 2007). Clinical studies have associated kallikreins with several types of cancers and especially hormone-associated carcinomas, such as breast, prostate and ovarian (Borgono *et al*, 2004; Borgono and Diamandis, 2004). The most recognised kallikrein is KLK3, also known as prostate-specific antigen, which is an established biomarker for detecting or monitoring prostate cancer progression and response to treatment (Magklara *et al*, 1999; Stephan *et al*, 2002).

Kallikreins 4, 5, 6, 7, 8, 9, 10, 11, 13, 14 and 15 are highly expressed in ovarian cancer patients or in ovarian cancer cell lines, at the mRNA and/or protein level (Tanimoto *et al*, 1999; Shvartsman *et al*, 2003; Borgono *et al*, 2004; Lu *et al*, 2004; Ni *et al*, 2004; Santin *et al*, 2004). Four recent studies verified the overexpression of several kallikreins in ovarian cancer tissues (Yousef *et al*, 2003), in disseminated ovarian cancer cells isolated from ascites fluid of ovarian cancer patients (Oikonomopoulou *et al*, 2006b) and in ovarian cancer effusions (Shaw and Diamandis, 2007; Shih *et al*, 2007). Some kallikreins, such as KLK6 (Hoffman *et al*, 2002) and KLK10 (Luo *et al*, 2001), broadcast unfavourable prognosis, whereas others, such as KLK8 (Borgono *et al*, 2006), have been associated with favourable prognosis.

In this study, we investigated the serum levels of kallikreins 5, 6, 7, 8, 10 and 11 in relation to the clinical response of ovarian cancer patients to chemotherapy, the survival outcome (overall and progression-free survival), as well as the time to progression (TTP) of the disease. We also included the novel cancer markers B7-H4 (Simon *et al*, 2007a, 2007b), regenerating protein IV (Reg-IV) and Spondin-2 (Simon *et al*, 2007b), which have been reported to be differentially expressed in ovarian cancer. We performed multiparametric analysis, including or excluding CA125, in an effort to improve the predictive and/or prognostic value of this panel for ovarian cancer.

## Materials and methods

### Ovarian cancer patients and specimens

Ninety-eight patients with primary epithelial ovarian cancer were included in this study, ranging in age from 22 to 77 years, with a median of 50 years. Patients with stage I–IV ovarian carcinomas (FIGO stage I: *n*=14; stage II: *n*=5; stage III: *n*=73; stage IV: *n*=6) were included in this study. Patients received the adjuvant chemotherapy regimen CBDCA/CFA or Taxol/CBDCA.

Among the 95 patients with known clinical response, according to WHO criteria, 58 (61%) patients had good response, categorised as complete remission (CR, *n*=29), partial remission (PR, *n*=21) or stable disease (SD, *n*=8) and 37 patients had poor clinical response with progressive disease (PD). Among the 50 CR and PR patients, 27 experienced disease progression, with a median TTP of 6 months (ranging from 3 to 24 months). Patients were monitored for survival and disease progression with a median follow-up of 24 months (ranging from 2 to 36 months). For 45 patients, the final end point within the time limits of this study was death.

Blood was drawn before the first chemotherapy and centrifuged within an hour for serum collection. Serum samples were stored at −80 °C until analysis. CA125 values were measured for all patients, utilising a Roche Elecsys method (Roche, Mississauga, ON, Canada).

### Quantification of biomarkers in serum

We quantified protein levels of KLKs 5, 6, 7, 8, 10 and 11, as well as B7-H4, Reg-IV and Spondin-2, in the serum of ovarian cancer patients by sandwich-type enzyme-linked immunosorbent assays, as previously described (Supplementary Table 1, Simon *et al*, 2007a, 2007b and our unpublished data). KLKs were quantified in Toronto (Dr Eleftherios P, Diamantis, Mount Sinai Hospital, Toronto, ON, Canada) and the other three biomarkers at diaDexus Inc. (San Francisco, CA, USA). All assays are highly sensitive and specific for each of the measured biomarkers.

### Statistical and clinical evaluation of the results

#### End points

For our analysis, we considered four end points: (a) clinical response, which was subcategorised as good (CR, PR, SD) *vs* poor response (PD); (b) overall survival, defined as time from first chemotherapy to death from any cause; (c) progression-free survival, defined as time from first chemotherapy to any progression or death from any cause; and (d) time from the first chemotherapy to progression (TTP) among CR and PR patients. For TTP, death was a competing risk and was treated as censored data.

#### Biomarkers

Serum samples were obtained before therapy (baseline specimen; c_0_) and before each chemotherapy cycle (c_1_–c_n_). The markers B7-H4, Spondin-2 and Reg-IV were measured only at c_0_, due to limited sample availability. As 59 patients had at least two measurements for each marker and 32 patients had at least three measures available, we considered marker concentration before and after the first treatment cycle (c_0_, c_1_) in logarithmic scale, relative changes in tumour marker after the first and the second cycle of chemotherapy (rc_1_=log (c_0_/c_1_); rc_2_=log (c_1_/c_2_)) and sometimes the ratios of these changes, (rc_1_/rc_2_), when sample sizes were deemed sufficient.

The relationships between biomarkers with patient and tumour characteristics were examined with the Kruskal–Wallis test, a non-parametric method for examining differences among multiple groups. Spearman's rank correlation coefficient was utilised to assess the correlations among biomarkers. The primary outcome for survival analyses was the progression-free survival, defined as the time from diagnosis to ovarian cancer recurrence or death from any cause. Patients, who were alive and did not meet any events, as defined by these end points, were censored at the time the last vital status was ascertained. Kaplan–Meier curves were utilised to present the survival probabilities as a function of time among groups of patients, defined by the tertile of the marker values, and log-rank tests were utilised to examine the overall difference among the curves. Cox regression model was applied to evaluate the hazard ratios (HRs) of biomarkers on overall and progression-free survival. Clinical parameters, including age and stage of disease, were adjusted in multivariate Cox proportional hazard models. Logistic regression was performed to calculate the odds ratio (OR) that defines the relation between biomarkers and response to therapy, where the outcome is response (partial response or complete response) *vs* no response (no change or progression). Both HR and OR were calculated on log-transformed biomarkers and were represented with their 95% confidence interval (95% CI) and two-sided *P*-values.

To further evaluate the diagnostic or prognostic usefulness of the markers for dichotomous classification, we considered receiver-operating characteristic (ROC) curve analysis. If by convention larger values of a biomarker were associated with adverse outcome, a cutoff point was utilised to define a positive marker-based test result, that is positive if the marker value exceeded a certain cutoff point. For a marker measured on continuous scales, the ROC curve was evaluated for all possible cutoff points. For binary outcome, that is response to chemotherapy, the ROC curve quantified the discriminatory ability of a marker for distinguishing between responders and non-responders. For TTP analysis, where the disease outcome is not concurrent with the test and the accuracy is a function of time, time-dependent ROC techniques for censored survival times were considered (Heagerty *et al*, 2000). We compared the true positive fraction, *P* (marker>cutoff point; death within *t* year) and false positive fraction, *P* (marker>cutoff point; survived beyond *t* year), across all possible cutoff points, and for *t* equal to 1 year and 5 years, respectively. For each ROC curve, we calculated the area under the curve (AUC), which ranges from 0.5 (for a non-informative marker) to 1 (for a perfect marker) and corresponds to the probability that a randomly selected patient who dies within *t* years has a higher marker value than a randomly selected patient who survived. Bootstrap method was utilised to calculate the CIs for AUC.

The ROC analysis was first conducted on individual markers and then in combination to explore the potential that a panel of markers can provide improved performance. We considered an algorithm that renders a single composite score using the linear predictor fitted from a binary regression model. This algorithm has been justified to be optimal under the linearity assumption (Eguchi and Copas, 2002; McIntosh and Pepe, 2002) in the sense that ROC curve is maximised (i.e., best sensitivity) at every threshold value. In particular, a weighted logistic regression which is appropriate for censored failure time data was utilised (Zheng *et al*, 2006) for deriving the prognostic index. A stepwise regression procedure was utilised to select markers within the panel, sometimes along with clinical variables.

As an independent validation series was not available for this study, the predictive accuracy of the composite scores was evaluated based on re-sampling of the original data. Specifically, we randomly split the data into a learning set and a test set. The learning set included two of three of the observations and the test set one of three of the observations. Using the learning set, we first performed model selection from which the final selected model gave rise to the linear combination rule. We then calculated two ROC curves for the linear score, one using data from the learning set and the other from the training set. The vertical differences between the two ROC curves gave the overestimation of the sensitivities at given specificities. The whole procedure was repeated 200 times and these differences were averaged to yield an estimate of the expected overestimation. For each type of the ROC analyses mentioned above, we present both the original ROC curves and the ROC curves that are corrected for overestimation.

All statistical analyses were performed using software SAS 9.1 (SAS Institute Inc., Cary, NC, USA) and S-PLUS 7.0 (Insightful Corp., Seattle, WA, USA).

## Results

### Associations of biomarkers with clinicopathological factors

Significant correlations (*P*<0.05) were observed between most of the biomarkers, in particular among CA125 and all markers except KLK7 (Supplementary Table 2). Supplementary Table 3 presents distributions of individual markers, stratified by age and stage. Lower levels of CA125, B7-H4 and Spondin-2 were significantly associated with older age (all *P*<0.05), whereas higher values of markers tended to be associated with higher clinical stages for CA125, KLK5, KLK6, KLK10, KLK11, B7-H4 and Spondin-2 (all *P*<0.05).

### Associations of biomarkers with response to chemotherapy

We considered the association of serially measured biomarkers with clinical response to chemotherapy (good: CR, PR or SD *vs* poor: PD) using logistic regression models (Table 1). Univariately, statistically significant predictors of response included baseline measures of CA125, KLK6, KLK8, KLK10, B7-H4 and Reg-IV (all *P*<0.05), with a lower baseline level of the marker associated with favourable response. In addition, changes in CA125 from first chemotherapy cycle compared to baseline also appeared to predict response (OR=1.84, 95% CI (1.12, 3.03), *P*=0.017). Similarly, rapid decreases in KLK5 level after the first chemotherapy cycle also appeared to associate with favourable response (OR=1.86, 95% CI (1.09, 3.18), *P*=0.022). As age and stage were not significantly related to chemotherapy response, we did not consider further multivariate regression models adjusting for age or stage of disease.

**Figure 1:**
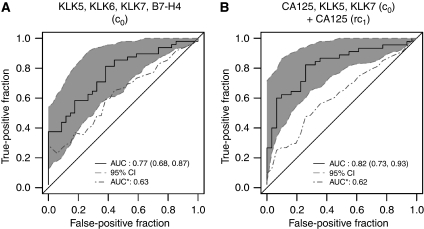
ROC curves for chemotherapy response using marker panels. ROC curves for the ‘combined’ marker, without (‘original’, solid line) and with correction for overfitting (‘corrected’, dashed line). The correction for overfitting was done by the cross-validation procedure described under Materials and methods. (**A**) Combination using only baseline values (c_0_) of KLK5, KLK6, KLK7 and B7-H4. (**B**) Combination using baseline values (c_0_) of CA125, KLK5 and KLK7 and relative changes (rc_1_=log (c_0_/c_1_)) of CA125 from baseline to the first chemotherapy. Data for good *vs* poor response are further described in Table 1 and the combined model is described in Supplementary Table 4.

Receiver-operating characteristic curve analysis was utilised to evaluate the clinical usefulness of these markers for predicting response to chemotherapy. When considered separately, these markers only had moderate discriminatory capacity for separating good from poor responders. For example, the baseline level of KLK6 had an AUC of 0.69 (95% CI (0.58, 0.80)) (Table 1), whereas changes of CA125 after the first cycle had an AUC of 0.69 (95% CI (0.58, 0.81)). We further explored if the combination of a panel of markers could improve this diagnostic accuracy. We employed a stepwise model selection procedure that took all the baseline markers into consideration. Using the linear predictors from the final model involving KLK5, KLK6, KLK7 and B7-H4, we obtained an ROC curve with an AUC of 0.77 (95% CI (0.68, 0.87)). However, due to sample-size limitation, when correcting for the uncertainty in both model selection and ROC-estimation cross-validation procedure, the analysis resulted in a corrected AUC of only 0.63 (Figure 1A). We also considered building a logistic regression model using information on both baseline concentration and relative changes after each chemotherapy cycle. The final selected multivariate model, which included baseline measures of CA125, KLK5 KLK7 and the relative change at the first chemotherapy for CA125, resulted in an ROC curve that had an AUC of 0.82 (95% CI (0.73, 0.93)). However, due to limited sample size and even greater uncertainty involved in the model-fitting procedure, the corrected AUC was 0.62 (Figure 1B). Therefore, with the current sample size, we cannot conclude if including longitudinal information can improve the predictive accuracy.

### Associations of biomarkers with overall survival

The Kaplan–Meier estimates of time to overall survival for patients stratified in three risk groups, as defined by tertiles of the level of an individual marker, indicated that many of the markers have good prognostic values (Supplementary Figure 1). In addition, we also saw good separations of Kaplan–Meier curves when patients were classified by both stage (I/II *vs* III/IV) and marker concentrations (upper, defined as >median value; *vs* lower, defined as <median value) (Supplementary Figure 1), suggesting that these markers may provide useful prognostic information in addition to clinical staging criteria.

The unadjusted and adjusted HRs estimated from Cox proportional hazard models are presented in Table 2. Higher values of baseline measures of CA125, KLK5, KLK6, KLK10, B7-H4 and Spondin-2 were all significantly associated with worse overall survival. For example, the HR for Spondin-2 was 2.67 (95% CI (1.43, 4.96), *P*=0.002). The prognostic values of many of the kallikreins, B7-H4 and Spondin-2 were stronger than those of CA125 (based on HR values), which had a HR of 1.34 (95% CI (1.17, 1.53), *P*<0.001). Adjustment for clinical variables including age, stage and response to chemotherapy was also performed. When considering this multivariate model, some markers became insignificant but some of them retained their significance. For example, an HR of 1.28 (95% CI (1.02, 1.60), *P*=0.034) was observed for KLK5 and 1.69 (95% CI (1.14, 2.49), *P*=0.008) for B7-H4, indicating that these two markers may provide additional prognostic value beyond age, clinical stage or response to chemotherapy. Changes in some marker concentrations after each chemotherapy treatment cycle also appeared to predict survival. In particular, rc_1_ of KLK6 significantly predicted time to survival in the univariate Cox regression models (HR=1.77, 95% CI (1.07, 2.95), *P*=0.028); however, none of the subsequent measurements remained a significant independent predictor in the multivariate model (with the exception of KLK6 rc_1_/rc_2_ ratio).

**Figure 2:**
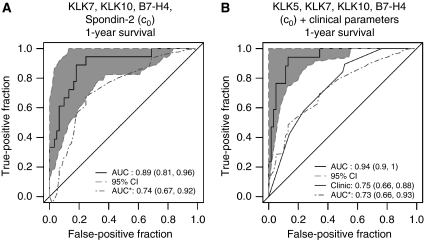
ROC curves for overall survival at 1-year post-chemotherapy, using a panel of biomarkers, without (‘original’, solid line) and with correction for overfitting (‘corrected’, dashed line). The correction for overfitting was done by the cross-validation procedure described under Materials and methods. Data for overall survival are further described in Table 2 and the combined model is described in Supplementary Table 4. (**A**) Combination using baseline values (c_0_) of KLK7, KLK10, B7-H4 and Spondin-2. (**B**) Combination using baseline values (c_0_) of KLK5, KLK7, KLK10 and B7-H4 and the clinical parameters age, stage and response to chemotherapy.

To assess whether the markers have good capacity in discriminating between subjects who progress before a given time *t* and those who survive beyond *t*, the time-dependent ROC method was utilised to evaluate the prognostic accuracy of the biomarkers. We plotted ROC curves at 1 year to investigate whether they have the accuracy to separate short-term survivors (failed by 1 year *vs* alive beyond 1 year). Although univariately, the predictive accuracy of each individual marker was quite low (data not shown), it was interesting to see whether we could identify a marker panel that would offer improved prognostic accuracy. We utilised a weighted logistic regression for survival data that aimed to directly find a linear combination of markers with which the area under the time-dependent ROC curve is maximised at each *t*. We found that a combination of markers may have the potential for improved prognostic accuracy. A linear combination rule considering KLK7, KLK10, B7-H4 and Spondin-2 yielded an ROC curve for survival at 1 year after chemotherapy with an AUC of 0.89 (95% CI (0.81, 0.96)) (Figure 2A). After correcting for overfitting, the prognostic performance was retained with an AUC of 0.74. Addition of clinical data (age, stage and response to chemotherapy) to the selected marker panel further improved the unadjusted AUC to 0.94 (Figure 2B). No improvement was observed for 2-year mortality even with a combination of a panel of markers (data not shown). These results suggest that there may be a good potential for the panel of markers to achieve good short-term (within 1-year) prognostic performance.

### Associations of biomarkers with progression-free survival

We performed Kaplan–Meier analysis of TTP for our patient groups, concluding that many of the markers have prognostic values (Supplementary Figure 2). The curves also showed a good separation when patients were divided into groups, according to marker concentrations (upper, defined as >median value; *vs* lower, defined as <median value).

The unadjusted HRs for progression-free survival are listed in Table 3. Higher values of baseline measures of CA125, KLK5, KLK6, KLK10, KLK11, B7-H4 and Spondin-2 were all significantly associated with worse progression-free survival. For example, the HR for Spondin-2 was 2.21 (95% CI (1.3, 3.76), *P*=0.003). The prognostic values of many of the kallikreins, as well as of the markers B7-H4 and Spondin-2, were stronger than those of CA125 (based on HR values). Adjustment for clinical variables, including age, stage and response to chemotherapy, resulted in HRs of 1.23 (95% CI (1.07, 1.42), *P*=0.004) for CA125, 1.33 (95% CI (1.08, 1.64), *P*=0.008) for KLK5, 1.44 (95% CI (1.06, 1.97), *P*=0.022) for KLK10, 1.67 (95% CI (1.10, 2.55), *P*=0.017) for KLK11 and 1.71 (95% CI (1.25, 2.35), *P*=0.001) for B7-H4. Changes in marker concentrations after the chemotherapy cycles also appeared to predict progression-free survival. In particular, rc_1_ of CA125, KLK10 and KLK11 significantly predicted TTP in the multivariate Cox regression models (all *P*-values <0.05) that adjust for age, stage and chemotherapy response, indicating that decreases in these markers after the first chemotherapy cycle may be associated with an adverse event in the future.

We further evaluated the prognostic accuracy for different risk classifications of a panel of biomarkers. A linear combination of some markers (CA125, KLK7, KLK8 and KLK10 at the baseline) resulted in an ROC curve at 1 year, after initiation of treatment, with AUC 0.77 (95% CI (0.70, 0.90)). However, when corrected for overfitting, the AUC for corrected ROC curve was reduced to 0.62 (data not shown).

### Associations of biomarkers with TTP among CR and PR patients

**Figure 3:**
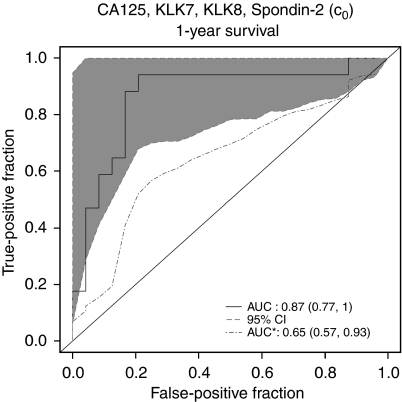
ROC curves for time to progression among CR and PR patients using a panel of biomarkers. Presented is the original ROC curve (solid line) with its 95% confidence interval (shaded area) and the ROC curve calculated using cross-validation to correct for overfitting (AUC with an asterisk, dashed line). Combined markers for included baseline measures (c_0_) of CA125, KLK7, KLK8 and Spondin-2 are further described in Supplementary Table 4.

For the 50 patients who were in CR (*n*=29) or PR (*n*=21), we examined whether the markers can predict their times to progression. We investigated this question by analysing the predictive value for time to cancer progression for each individual biomarker. The results of the Cox analyses are summarised in Table 4. Younger (<50 years old) or later stage (stages III and IV) patients were associated with higher risk of progression, although the effect of age was only marginally significant. Except for KLK7 and Reg-IV, higher concentrations of all other markers at baseline were associated with higher risk of progression. For example, CA125 had an HR of 1.71 (95% CI (1.37, 2.13), *P*<0.001), whereas KLK5 had an HR of 2.44 (95% CI (1.59, 3.75), *P*<0.001) and Spondin-2 had an HR of 3.35 (95% CI (1.54, 7.26), *P*=0.002). In addition, changes after first chemotherapy from baseline of CA125, KLK5, KLK10 and KLK11 were significant predictors for TTP. Combining CA125, KLK7, KLK8 and Spondin-2 at baseline yielded good prognostic accuracy for predicting patients with progression by 1 year: the area under the ROC curve at 1 year after treatment was 0.87 (95% CI (0.77, 1.00)), but was reduced to 0.65 when potential overfitting was adjusted (Figure 3). Although there is potential that the prognostic accuracy could be further improved using longitudinal measures, potential of overfitting is also significant, given our sample size. We therefore did not explore this further.

In summary, the regression models utilised for ROC curve analysis for a panel of markers with different outcomes are presented in Supplementary Table 4.

## Discussion

Ovarian cancer detection, prognosis and response to treatment are currently based on quantification of serum levels of CA125 and imaging modalities (Van der Berg *et al*, 1988; Marsden *et al*, 2000; Meyer and Rustin, 2000). However, the clinical value of CA125 as a marker of ovarian cancer is low, due to its expression in non-malignant or non-ovarian malignant conditions (Tuxen *et al*, 1995; Meyer and Rustin, 2000). Serum CA125 is presently considered as a marker of response to treatment rather than a screening/diagnostic marker (Van der Berg *et al*, 1988; Tuxen *et al*, 1995; Rustin *et al*, 1996; Zanotti and Kennedy, 1999). As part of the quest to identify useful biomarkers for detection and management of ovarian carcinoma, the idea of multimarker analysis has been adopted by many groups (Bose and Mukherjea, 1994; Zhang *et al*, 2004). Thus far, increases in the diagnostic sensitivity of CA125 by using marker panels have been moderate (5–10%), often at the expense of decreases in specificity, and vice versa (Tuxen *et al*, 1995; Meyer and Rustin, 2000; Bast, 2003; Zhang *et al*, 2004).

Members of one family of cancer-related markers, the kallikrein serine protease family, have been associated with either favourable or unfavourable prognostic value in ovarian cancer or they are candidate diagnostic markers (Borgono *et al*, 2004, 2006; Borgono and Diamandis, 2004; Shan *et al*, 2006; McIntosh *et al*, 2007). The clinical applicability of the other three candidate ovarian cancer markers included in this study, B7-H4 (Simon *et al*, 2007a, 2007b), Reg-IV and Spondin-2 (Simon *et al*, 2007b), is still unknown.

We found significant correlations between the serological markers used in this study. For example, CA125 correlated significantly with all the markers studied, except KLK7 (Supplementary Table 2). These data are in agreement with previous observations (Borgono *et al*, 2004; Borgono and Diamandis, 2004). Utilisation of such biomarkers in multiparametric analyses may prove beneficial for ovarian cancer diagnosis, prognosis and prediction of therapeutic response. As a proof of this principle, in a recent study, a panel of kallikreins (KLKs 4, 5, 6, 7, 8, 10, 11, 13 and 14) was able to confidently discriminate cancerous from benign effusions, with an area under the ROC curve of 0.99, and ovarian cancer from other cancer groups with an AUC of 0.96 (Shih *et al*, 2007).

In our study, the predictive power of many markers for overall (Table 2) and progression-free survival (Table 3) at univariate analysis was encouraging. For example, CA125, KLKs 5, 6, 10, B7-H4 and Spondin-2 baseline values (c_0_) predicted a worse overall survival (Table 2) and CA125, KLKs 5, 6, 10, 11, B7-H4 and Spondin-2 baseline values (c_0_) a worse progression-free survival of ovarian cancer patients (Table 3). Many of these markers were independent of clinical parameters, such as age, FIGO stage or response to treatment, when multivariate analysis was performed. Similarly to the previous study for ovarian cancer detection (Shih *et al*, 2007), we considered panels of these markers to predict patient survival. Such a panel, which included KLK7, KLK10, B7-H4 and Spondin-2 baseline values (c_0_), was significantly predictive of 1-year survival post-therapy, with an AUC of 0.89 (Figure 2A).

Moreover, our findings suggested that high baseline values (c_0_) of markers (CA125, KLKs 6, 8, 10, B7-H4 and Reg-IV), as well as changes of CA125 and KLK5 after the first chemotherapy cycle (rc_1_), individually predicted an unfavourable response to chemotherapy and a higher risk for disease progression (Table 1). A panel of markers comprising KLK5, KLK6, KLK7 and B7-H4 baseline levels (c_0_) resulted in an improved AUC of 0.77 for predicting response to chemotherapy (Figure 1A).

Baseline values (c_0_) of all markers, except of KLK7 and Reg-IV, were also significant predictors of TTP among the group of responders to chemotherapy (Table 4). Combination of CA125, KLK7, KLK8 and Spondin-2 baseline levels predicted patients who would progress within 1 year, with an ROC curve of 0.87 (Figure 3).

Notably, thirty-one of the patients included in this study had a baseline serum CA125 concentration that was close to or below the cutoff point of 30 U ml^−1^ and remained non-informative (no significant change) over the course of chemotherapy. For these 31 patients, a few of the examined markers, for example KLK6 (OR=0.42; *P*=0.004), KLK8 (OR=0.44; *P*=0.026), KLK10 (OR=0.59; *P*=0.03), B7-H4 (OR=0.60; *P*=0.03) and Reg-IV (OR=0.36; *P*=0.027) appeared to be associated with response to chemotherapy. These data suggest that some markers may be superior or complement CA125 for predicting response to chemotherapy treatment.

It has been reported that the chemopreventive agent *α*-difluoromethylornithine is not able to efficiently block expression of many tumorigenesis-related genes, including kallikrein 6, in activated Ki-ras-expressing Caco-2 cells (Ignatenko *et al*, 2004). In this regard, it is possible that kallikreins may have a function in chemotherapy-resistant tumours, as it has already been proposed for kallikreins 3 (Foekens *et al*, 1999) and 10 (Luo *et al*, 2002) in breast cancer and kallikrein 4 in ovarian cancer (Xi *et al*, 2004). A similar role has also been proposed for Reg-IV in gastric tumours (Mitani *et al*, 2007). The markers included in this study should be, therefore, validated with a larger sample size to establish their applicability for ovarian cancer prognosis and response to chemotherapy.

Amongst the markers evaluated in this study, the members of the kallikrein family have been reported to cleave extracellular matrix proteins associated with tissue remodelling (Borgono and Diamandis, 2004; Ghosh *et al*, 2004; Michael *et al*, 2005), as well as activate or disarm protease-activated receptors (Oikonomopoulou *et al*, 2006a, Ramachandran and Hollenberg, 2007). Furthermore, they have been examined as putative modulators of neovascularisation (Aimes *et al*, 2003) and potential activators of molecules associated with tumour growth, survival, invasion or metastasis (Yoi *et al*, 1979; Frenette *et al*, 1997; Takayama *et al*, 2001; Petraki *et al*, 2002; Borgono and Diamandis, 2004). In this regard, kallikreins may be valuable targets for developing novel cancer therapeutic interventions.

## Figures and Tables

**Table 1:** Univariate logistic regression for chemotherapy response (good *vs* poor)

**Clinical variable**	***N* (%)**	**OR**	**CI**	***P***	
*Age (years)*					
⩽50	52 (54%)	1			
>50	45 (46%)	1.09	(0.47, 2.51)	0.842	
					
*Stage*					
I or II	19 (19%)	1			
III or IV	79 (81%)	0.89	(0.32, 2.53)	0.833	
					
	**Poor**		**Good**		
**Markers**	***N***	**Mean (s.d.)^a^**	***N***	**Mean (s.d.)**	*P* **
*CA125*					
Baseline (c_0_)	37	5.488 (2.227)	57	4.401 (1.854)	0.023
rc_1_=log(c_0_/c_1_)	34	0.121 (1.069)	53	0.753 (1.105)	0.003
rc_2_=log(c_1_/c_2_)	20	0.339 (0.473)	34	0.587 (1.191)	0.986
rc_1_/rc_2_	20	0.181 (1.215)	33	0.223 (1.338)	0.279
					
*KLK5*					
Baseline (c_0_)	37	−1.776 (1.629)	58	−1.657 (1.03)	0.466
rc_1_=log(c_0_/c_1_)	35	−0.141 (1.016)	54	0.372 (0.903)	0.016
rc_2_=log(c_1_/c_2_)	22	0.111 (0.482)	34	0.217 (0.584)	0.860
rc_1_/rc_2_	22	−0.141 (1.216)	34	0.134 (1.149)	0.140
					
*KLK6*					
Baseline (c_0_)	37	3.077 (0.808)	58	2.562 (0.743)	0.002
rc_1_=log(c_0_/c_1_)	35	0.175 (0.668)	53	0.084 (0.645)	0.932
rc_2_=log(c_1_/c_2_)	22	0.036 (0.568)	34	0.082 (0.466)	0.827
rc_1_/rc_2_	22	0.286 (1.191)	34	0.003 (0.927)	0.627
					
*KLK7*					
Baseline (c_0_)	37	1.283 (0.501)	58	1.071 (0.579)	0.0747
rc_1_=log(c_0_/c_1_)	35	0.013 (0.374)	53	0.012 (0.537)	0.956
rc_2_=log(c_1_/c_2_)	22	0.016 (0.291)	34	0.078 (0.319)	0.568
rc_1_/rc_2_	22	0.01 (0.581)	34	−0.16 (0.557)	0.450
					
*KLK8*					
Baseline (c_0_)	37	2.54 (0.653)	58	2.246 (0.569)	0.017
rc_1_=log(c_0_/c_1_)	35	0.062 (0.466)	53	0.019 (0.511)	0.673
rc_2_=log(c_1_/c_2_)	22	−0.013 (0.405)	34	0.114 (0.364)	0.302
rc_1_/rc_2_	22	0.07 (0.737)	34	−0.15 (0.717)	0.227
					
*KLK10*					
Baseline (c_0_)	37	1.463 (0.864)	58	1.022 (0.947)	0.013
rc_1_=log(c_0_/c_1_)	35	0.126 (0.511)	53	0.198 (0.74)	0.805
rc_2_=log(c_1_/c_2_)	22	0.073 (0.218)	34	0.076 (0.351)	0.481
rc_1_/rc_2_	22	0.073 (0.709)	34	0.108 (0.7)	0.663
					
*KLK11*					
Baseline (c_0_)	36	0.052 (0.774)	57	−0.199 (0.737)	0.196
rc_1_=log(c_0_/c_1_)	34	0.001 (0.632)	53	0.192 (0.677)	0.058
rc_2_=log(c_1_/c_2_)	22	0.129 (0.358)	34	0.066 (0.306)	0.574
rc_1_/rc_2_	21	0.03 (0.901)	34	0.176 (0.527)	0.161
					
*B7-H4*					
Baseline (c_0_)	35	1.216 (1.006)	52	0.719 (0.976)	0.009
*Reg-IV*					
Baseline (c_0_)	34	−0.246 (0.517)	49	−0.518 (0.521)	0.027
					
*Spondin-2*					
Baseline (c_0_)	34	4.398 (0.551)	49	4.209 (0.542)	0.101

CI=confidence interval; OR=odds ratio.

a Logarithmically transformed values.

** Wilcoxon Rank Sum test.

**Table 2:** Cox regression for overall survival outcome

		**Univariate**			**Multivariate**		
**Clinical variable**	***N* (%)**	**HR**	**CI**	***P***	**HR**	**CI**	***P***
*Age (years)*							
⩽50	52 (54%)	1			1		
>50	45 (46%)	0.56	(0.3, 1.06)	0.074	0.63	(0.33, 1.21)	0.166
							
*Stage*							
I or II	19 (19%)	1			1		
III or IV	79 (81%)	2.49	(0.98, 6.34)	0.056	2.04	(0.78, 5.36)	0.146
							
*Chemotherapy response*							
Poor	37 (37%)	1			1		
Good	58 (61%)	0.18	(0.09, 0.35)	<0.001	0.19	(0.1, 0.37)	<0.001
							
**Markers**							
*CA125*							
Baseline (c_0_)	97	1.34	(1.17, 1.53)	<0.001	1.16	(0.98, 1.38)	0.086
rc_1_=log (c_0_/c_1_)	90	0.96	(0.71, 1.31)	0.792	1.06	(0.75, 1.51)	0.732
rc_2_=log (c_1_/c_2_)	57	1.36	(0.94, 1.96)	0.102	1.49	(0.94, 2.36)	0.087
rc_1_/rc_2_	56	1.01	(0.69, 1.47)	0.967	1.08	(0.73, 1.6)	0.685
							
KLK5							
Baseline (c_0_)	98	1.33	(1.03, 1.71)	0.028	1.28	(1.02, 1.6)	0.034
rc_1_=log (c_0_/c_1_)	92	0.91	(0.64, 1.27)	0.571	1.04	(0.73, 1.48)	0.827
rc_2_=log (c_1_/c_2_)	59	1.66	(0.87, 3.16)	0.125	1.63	(0.76, 3.51)	0.208
rc_1_/rc_2_	59	0.95	(0.68, 1.34)	0.780	1.06	(0.74, 1.53)	0.748
							
*KLK6*							
Baseline (c_0_)	98	2.09	(1.4, 3.11)	<0.001	1.36	(0.88, 2.12)	0.168
rc_1_=log (c_0_/c_1_)	91	1.77	(1.07, 2.95)	0.028	1.49	(0.85, 2.6)	0.160
rc_2_=log (c_1_/c_2_)	59	0.89	(0.38, 2.08)	0.786	0.53	(0.24, 1.19)	0.125
rc_1_/rc_2_	59	1.57	(1.01, 2.45)	0.044	1.61	(1.08, 2.4)	0.020
							
*KLK7*							
Baseline (c_0_)	98	0.86	(0.5, 1.48)	0.595	0.59	(0.33, 1.08)	0.088
rc_1_=log (c_0_/c_1_)	91	0.9	(0.46, 1.75)	0.753	0.86	(0.39, 1.91)	0.713
rc_2_=log (c_1_/c_2_)	59	0.54	(0.15, 2.01)	0.359	0.71	(0.19, 2.7)	0.614
rc_1_/rc_2_	59	1.51	(0.7, 3.26)	0.289	1.25	(0.58, 2.68)	0.570
							
*KLK8*							
Baseline (c_0_)	98	1.58	(0.95, 2.64)	0.080	1.16	(0.69, 1.94)	0.586
rc_1_=log (c_0_/c_1_)	91	1.13	(0.57, 2.23)	0.727	0.97	(0.48, 2)	0.942
rc_2_=log (c_1_/c_2_)	59	0.66	(0.23, 1.9)	0.443	1.48	(0.47, 4.62)	0.503
rc_1_/rc_2_	59	1.33	(0.78, 2.28)	0.289	0.99	(0.55, 1.78)	0.975
							
*KLK10*							
Baseline (c_0_)	98	1.47	(1.11, 1.96)	0.008	1.3	(0.89, 1.9)	0.176
rc_1_=log (c_0_/c_1_)	91	1.04	(0.65, 1.66)	0.880	1.13	(0.64, 2.01)	0.669
rc_2_=log (c_1_/c_2_)	59	1.08	(0.3, 3.89)	0.901	0.57	(0.11, 2.95)	0.505
rc_1_/rc_2_	59	1.31	(0.67, 2.54)	0.432	1.56	(0.83, 2.93)	0.169
							
*KLK11*							
Baseline (c_0_)	95	1.46	(0.93, 2.28)	0.097	1.18	(0.72, 1.91)	0.513
rc_1_=log (c_0_/c_1_)	89	0.98	(0.61, 1.57)	0.917	1.07	(0.63, 1.79)	0.811
rc_2_=log (c_1_/c_2_)	59	1.38	(0.45, 4.18)	0.572	1.09	(0.36, 3.33)	0.881
rc_1_/rc_2_	57	1.14	(0.64, 2.04)	0.659	1.22	(0.73, 2.03)	0.453
							
*B7-H4*							
Baseline (c_0_)	89	2.04	(1.48, 2.81)	<0.001	1.69	(1.14, 2.49)	0.008
							
*Reg-IV*							
Baseline (c_0_)	85	1.56	(0.89, 2.74)	0.119	1	(0.52, 1.92)	0.996
							
*Spondin-2*							
Baseline (c_0_)	85	2.67	(1.43, 4.96)	0.002	1.65	(0.81, 3.35)	0.167

CI=confidence interval; HR=hazard ratio.

**Table 3:** Cox regression for progression-free survival outcome

		**Univariate**			**Multivariate**		
**Clinical variable**	***N* (%)**	**HR**	**CI**	***P***	**HR**	**CI**	***P***
*Age (years)*							
⩽50	52 (54%)	1			1		
>50	45 (46%)	0.59	(0.35, 1)	0.049	0.68	(0.4, 1.17)	0.166
							
*Stage*							
I or II	19 (19%)	1			1		
III or IV	79 (81%)	3.43	(1.47, 8.01)	0.004	2.98	(1.26, 7.03)	0.013
							
*Chemotherapy response*							
Poor	37 (37%)	1			1		
Good	58 (61%)	0.56	(0.33, 0.94)	0.030	0.62	(0.37, 1.06)	0.079
							
**Markers**							
*CA125*							
Baseline (c_0_)	97	1.31	(1.17, 1.46)	<0.001	1.23	(1.07, 1.42)	0.004
rc_1_=log (c_0_/c_1_)	90	1.4	(1.09, 1.78)	0.007	1.31	(1.01, 1.69)	0.042
rc_2_=log (c_1_/c_2_)	57	1.56	(1.18, 2.05)	0.002	1.47	(1.09, 1.98)	0.013
rc_1_/rc_2_	56	0.92	(0.67, 1.27)	0.605	0.94	(0.7, 1.28)	0.715
							
*KLK5*							
Baseline (c_0_)	98	1.41	(1.14, 1.74)	0.001	1.33	(1.08, 1.64)	0.008
rc_1_=log (c_0_/c_1_)	92	1.19	(0.9, 1.59)	0.224	1.16	(0.88, 1.54)	0.301
rc_2_=log (c_1_/c_2_)	59	2.16	(1.28, 3.63)	0.004	1.74	(0.97, 3.1)	0.062
rc_1_/rc_2_	59	0.95	(0.71, 1.27)	0.728	1.06	(0.78, 1.45)	0.701
							
*KLK6*							
Baseline (c_0_)	98	1.54	(1.11, 2.15)	0.011	1.23	(0.85, 1.77)	0.265
rc_1_=log (c_0_/c_1_)	91	1.56	(1.01, 2.39)	0.044	1.18	(0.74, 1.9)	0.491
rc_2_=log (c_1_/c_2_)	59	1.2	(0.54, 2.65)	0.659	0.8	(0.37, 1.72)	0.560
rc_1_/rc_2_	59	1.25	(0.83, 1.87)	0.292	1.32	(0.91, 1.89)	0.140
							
*KLK7*							
Baseline (c_0_)	98	1.19	(0.74, 1.91)	0.478	1.03	(0.64, 1.66)	0.897
rc_1_=log (c_0_/c_1_)	91	1.33	(0.73, 2.45)	0.354	1.28	(0.68, 2.4)	0.441
rc_2_=log (c_1_/c_2_)	59	0.69	(0.23, 2.09)	0.509	0.68	(0.22, 2.08)	0.497
rc_1_/rc_2_	59	1.32	(0.69, 2.53)	0.408	1.34	(0.7, 2.56)	0.376
							
*KLK8*							
Baseline (c_0_)	98	1.53	(0.98, 2.39)	0.061	1.33	(0.84, 2.11)	0.231
rc_1_=log (c_0_/c_1_)	91	1.3	(0.72, 2.35)	0.380	1.21	(0.65, 2.24)	0.549
rc_2_=log (c_1_/c_2_)	59	0.91	(0.37, 2.25)	0.834	1.28	(0.47, 3.5)	0.627
rc_1_/rc_2_	59	1.11	(0.69, 1.79)	0.664	1.05	(0.62, 1.78)	0.847
							
*KLK10*							
Baseline (c_0_)	98	1.6	(1.21, 2.12)	0.001	1.44	(1.06, 1.97)	0.022
rc_1_=log (c_0_/c_1_)	91	1.6	(1.12, 2.27)	0.010	1.56	(1.04, 2.33)	0.030
rc_2_=log (c_1_/c_2_)	59	3.06	(0.86, 10.87)	0.084	1.84	(0.46, 7.39)	0.391
rc_1_/rc_2_	59	1.07	(0.59, 1.94)	0.817	1.2	(0.67, 2.17)	0.536
							
*KLK11*							
Baseline (c_0_)	95	1.94	(1.33, 2.83)	0.001	1.67	(1.1, 2.55)	0.017
rc_1_=log (c_0_/c_1_)	89	1.7	(1.17, 2.46)	0.005	1.56	(1.04, 2.34)	0.031
rc_2_=log (c_1_/c_2_)	59	2.22	(0.91, 5.44)	0.081	1.5	(0.59, 3.84)	0.396
rc_1_/rc_2_	57	1.34	(0.81, 2.22)	0.248	1.43	(0.87, 2.35)	0.156
							
*B7-H4*							
Baseline (c_0_)	89	1.96	(1.49, 2.59)	<0.001	1.71	(1.25, 2.35)	0.001
							
*Reg-IV*							
Baseline (c_0_)	85	1.07	(0.65, 1.76)	0.804	0.89	(0.52, 1.53)	0.671
							
*Spondin-2*							
Baseline (c_0_)	85	2.21	(1.3, 3.76)	0.003	1.52	(0.83, 2.76)	0.173

CI=confidence interval; HR=hazard ratio.

**Table 4:** Cox regression for time to progression among CR and PR patients

**Clinical**		**Univariate**			**Multivariate**		
**variable**	***N* (%)**	**HR**	**CI**	***P***	**HR**	**CI**	***P***
*Age (years)*							
⩽50	27 (54%)	1					
>50	23 (46%)	0.47	(0.21, 1.05)	0.067	0.47	(0.21, 1.05)	0.065
*Stage*							
I or II	12 (24%)	1					
III or IV	38 (76%)	12.48	(1.69, 92.21)	0.013	12.52	(1.69, 92.57)	0.013
							
**Markers**							
*CA125*							
Baseline (c_0_)	49	1.71	(1.37, 2.13)	<0.001	1.52	(1.19, 1.93)	0.001
rc_1_=log(c_0_/c_1_)	47	1.87	(1.37, 2.56)	<0.001	1.54	(1.1, 2.14)	0.011
							
*KLK5*							
Baseline (c_0_)	50	2.44	(1.59, 3.75)	<0.001	2.07	(1.29, 3.32)	0.002
rc_1_=log(c_0_/c_1_)	48	1.5	(1.03, 2.19)	0.035	1.24	(0.87, 1.77)	0.239
							
*KLK6*							
Baseline (c_0_)	50	2.42	(1.36, 4.32)	0.003	1.95	(1.12, 3.41)	0.018
rc_1_=log(c_0_/c_1_)	47	1.6	(0.84, 3.05)	0.151	1.05	(0.53, 2.08)	0.882
							
*KLK7*							
Baseline (c_0_)	50	1.52	(0.76, 3.01)	0.233	1.37	(0.71, 2.65)	0.345
rc_1_=log(c_0_/c_1_)	47	1.37	(0.65, 2.88)	0.403	1.16	(0.52, 2.59)	0.721
							
*KLK8*							
Baseline (c_0_)	50	2.23	(1.09, 4.58)	0.028	1.69	(0.86, 3.31)	0.127
rc_1_=log(c_0_/c_1_)	47	1.31	(0.59, 2.92)	0.509	1.18	(0.48, 2.88)	0.719
							
*KLK10*							
Baseline (c_0_)	50	1.47	(1.02, 2.13)	0.040	1.47	(1.02, 2.13)	0.041
rc_1_=log(c_0_/c_1_)	47	1.58	(1.07, 2.32)	0.020	1.52	(0.96, 2.41)	0.071
							
*KLK11*							
Baseline (c_0_)	49	3.3	(1.85, 5.89)	<0.001	3.02	(1.54, 5.9)	0.001
rc_1_=log(c_0_/c_1_)	47	1.95	(1.26, 3.02)	0.003	1.7	(0.99, 2.93)	0.055
							
*OVR110*							
Baseline (c_0_)	45	1.89	(1.29, 2.78)	0.001	1.63	(1.11, 2.41)	0.014
							
*CLN101*							
Baseline (c_0_)	42	1.38	(0.65, 2.94)	0.398	1.4	(0.69, 2.82)	0.353
							
*PRO108*							
Baseline (c_0_)	42	3.35	(1.54, 7.26)	0.002	1.99	(0.87, 4.57)	0.103

CI=confidence interval; CR=complete remission; HR=hazard ratio; PR=partial remission.
