# Supplementary material for: Prediction of ovarian cancer prognosis and response to chemotherapy by a serum-based multiparametric biomarker panel
Source: Br J Cancer. 2008 Sep 2;99(7):1103–13. doi: 10.1038/sj.bjc.6604630 (PMC2567083; doi:10.1038/sj.bjc.6604630)

Supplementary Table 1: Immunofluorometric quantification of KLKs 5, 6, 7, 8, 10, 11.

| **KLK protein** | **Assay configuration^1^** | **Detection limit (μg/L)** |
| --- | --- | --- |
| KLK5 | Mono-mono | 0.05 |
| KLK6 | Mono-mono | 0.05 |
| KLK7 | Mono-mono | 0.2 |
| KLK8 | Mono-mono | 0.05 |
| KLK10 | Mono-mono | 0.02 |
| KLK11 | Mono-poly | 0.05 |

1. mono, mouse monoclonal antibody; poly, rabbit polyclonal antibody. For the detailed assay configurations the reader can consult Shaw and Diamandis, 2007.

**Supplementary Table 2: Spearman’s correlation coefficients**

|  | **CA125** | **KLK5** | **KLK6** | **KLK7** | **KLK8** | **KLK10** | **KLK11** | **B7-H4** | **Reg-IV** |
| --- | --- | --- | --- | --- | --- | --- | --- | --- | --- |
| **KLK5** | 0.48* |  |  |  |  |  |  |  |  |
| **KLK6** | 0.53* | 0.43* |  |  |  |  |  |  |  |
| **KLK7** | 0.09 | 0.31* | 0.36* |  |  |  |  |  |  |
| **KLK8** | 0.26* | 0.30* | 0.55* | 0.51* |  |  |  |  |  |
| **KLK10** | 0.48* | 0.50* | 0.65* | 0.37* | 0.47* |  |  |  |  |
| **KLK11** | 0.59* | 0.57* | 0.53* | 0.38* | 0.52* | 0.63* |  |  |  |
| **B7-H4** | 0.60* | 0.31* | 0.32* | 0.14 | 0.25* | 0.46* | 0.47* |  |  |
| **Reg-IV** | 0.23* | 0.05 | 0.44* | 0.09 | 0.19 | 0.14 | 0.24* | 0.14 |  |
| **Spondin-2** | 0.62* | 0.47* | 0.51* | 0.14 | 0.39* | 0.49* | 0.71* | 0.53* | 0.29* |

***: p < 0.05**

**Supplementary Table 3: Distribution of markers by clinical characteristics**

|  | **Age** | | |  | **Stage** | | |
| --- | --- | --- | --- | --- | --- | --- | --- |
|  | <=50 | >50 |  |  | I/II | III/IV |  |
|  | Median | Median | p-value |  | Median | Median | p-value |
| **CA125** | 5.64 | 3.58 | 0.01 |  | 2.71 | 5.38 | 0.002 |
| **KLK5** | -1.80 | -1.83 | 0.44 |  | -2.21 | -1.72 | 0.002 |
| **KLK6** | 2.72 | 2.52 | 0.35 |  | 2.29 | 2.77 | 0.004 |
| **KLK7** | 1.14 | 1.19 | 0.95 |  | 1.23 | 1.13 | 0.833 |
| **KLK8** | 2.35 | 2.43 | 1.00 |  | 2.24 | 2.43 | 0.213 |
| **KLK10** | 1.05 | 0.99 | 0.91 |  | 0.82 | 1.19 | 0.015 |
| **KLK11** | 0.00 | -0.32 | 0.29 |  | -0.60 | -0.06 | 0.001 |
| **B7-H4** | 0.82 | 0.40 | 0.03 |  | 0.42 | 0.85 | 0.014 |
| **Reg-IV** | -0.36 | -0.54 | 0.11 |  | -0.61 | -0.46 | 0.254 |
| **Spondin-2** | 4.51 | 4.11 | 0.03 |  | 3.84 | 4.45 | 0.001 |

1. Values represent logarithms (ln) of biomarker values.

2. p-values are from global nonparametric Kruskal-Wallis Test for testing the association between a marker and a clinical variable.

**Supplementary Table 4: Regression models utilized for ROC curve analysis for a panel of markers with different outcome**

|  | **Estimate** | **Standard Error** | **z value** | **Pr (>|z|)** |
| --- | --- | --- | --- | --- |
| **Regression model for response to chemotherapy (c_0_)** | | | | |
| (Intercept) | 5.72 | 1.60 | 3.58 | <0.001 |
| KLK5 (c_0_) | 0.68 | 0.27 | 2.49 | 0.01 |
| KLK6 (c_0_) | -0.97 | 0.40 | -2.43 | 0.01 |
| KLK7 (c_0_) | -0.90 | 0.50 | -1.78 | 0.08 |
| B7-H4 (c_0_) | -0.55 | 0.28 | -1.94 | 0.05 |
| **Regression model for response to chemotherapy (c_0_ + rc_1_)** | | | | |
| (Intercept) | 5.36 | 1.56 | 3.42 | <0.001 |
| CA125 (c_0_) | -0.58 | 0.19 | -3.14 | 0.002 |
| KLK5 (c_0_) | 0.60 | 0.31 | 1.96 | 0.05 |
| KLK7 (c_0_) | -1.47 | 0.60 | -2.44 | 0.01 |
| CA125 (rc_1_) | 1.05 | 0.34 | 3.06 | 0.002 |
| **Regression model for overall survival (c_0_)** | | | |  |
| (Intercept) | -7.65 | 3.57 | -2.14 | 0.03 |
| KLK7 (c_0_) | -1.91 | 0.62 | -3.07 | 0.002 |
| KLK10 (c_0_) | 1.22 | 0.47 | 2.56 | 0.01 |
| B7-H4 (c_0_) | 0.96 | 0.39 | 2.47 | 0.01 |
| Spondin-2 (c_0_) | 1.26 | 0.82 | 1.53 | 0.13 |
| **Regression model for overall survival (c_0_ + clinical parameters)** | | | | |
| (Intercept) | 7.17 | 4.03 | 1.78 | 0.08 |
| KLK5 (c_0_) | 0.94 | 0.46 | 2.05 | 0.04 |
| KLK7 (c_0_) | -3.66 | 1.15 | -3.18 | 0.002 |
| KLK10 (c_0_) | 1.85 | 0.70 | 2.64 | 0.01 |
| B7-H4 (c_0_) | 0.73 | 0.44 | 1.69 | 0.09 |
| age group | -1.83 | 0.90 | -2.03 | 0.04 |
| stage | -1.89 | 1.50 | -1.26 | 0.21 |
| chemotherapy response | -1.92 | 0.98 | -1.96 | 0.05 |
| **Regression model for time to progression** | | | |  |
| (Intercept) | -3.74 | 2.78 | -1.35 | 0.18 |
| CA125 (c_0_) | 0.94 | 0.27 | 3.44 | <0.001 |
| KLK7 (c_0_) | -1.07 | 0.76 | -1.42 | 0.16 |
| KLK8 (c_0_) | 2.56 | 1.01 | 2.54 | 0.01 |
| Spondin-2 (c_0_) | -1.37 | 0.90 | -1.52 | 0.13 |

**Legends for Supplementary Figures**

**Supplementary Figure 1:** Overall survival curves for markers among four groups defined by median of marker levels (high versus low) and clinical stage (I/II versus III/IV). P values were calculated by log-rank tests. c_0_ represents the baseline biomarker concentration and rc_1_ represents the relative changes after the first cycle of chemotherapy [rc_1_ = log (c_0_/c_1_)].

**Supplementary Figure 2:** Progression-free survival curves for markers among two groups defined by medians of marker levels (lower < median; upper > median). P values were calculated by log-rank tests. c_0_ represents the baseline biomarker concentration and rc_1_ represents the relative changes after the first cycle of chemotherapy [rc_1_ = log (c_0_/c_1_)].


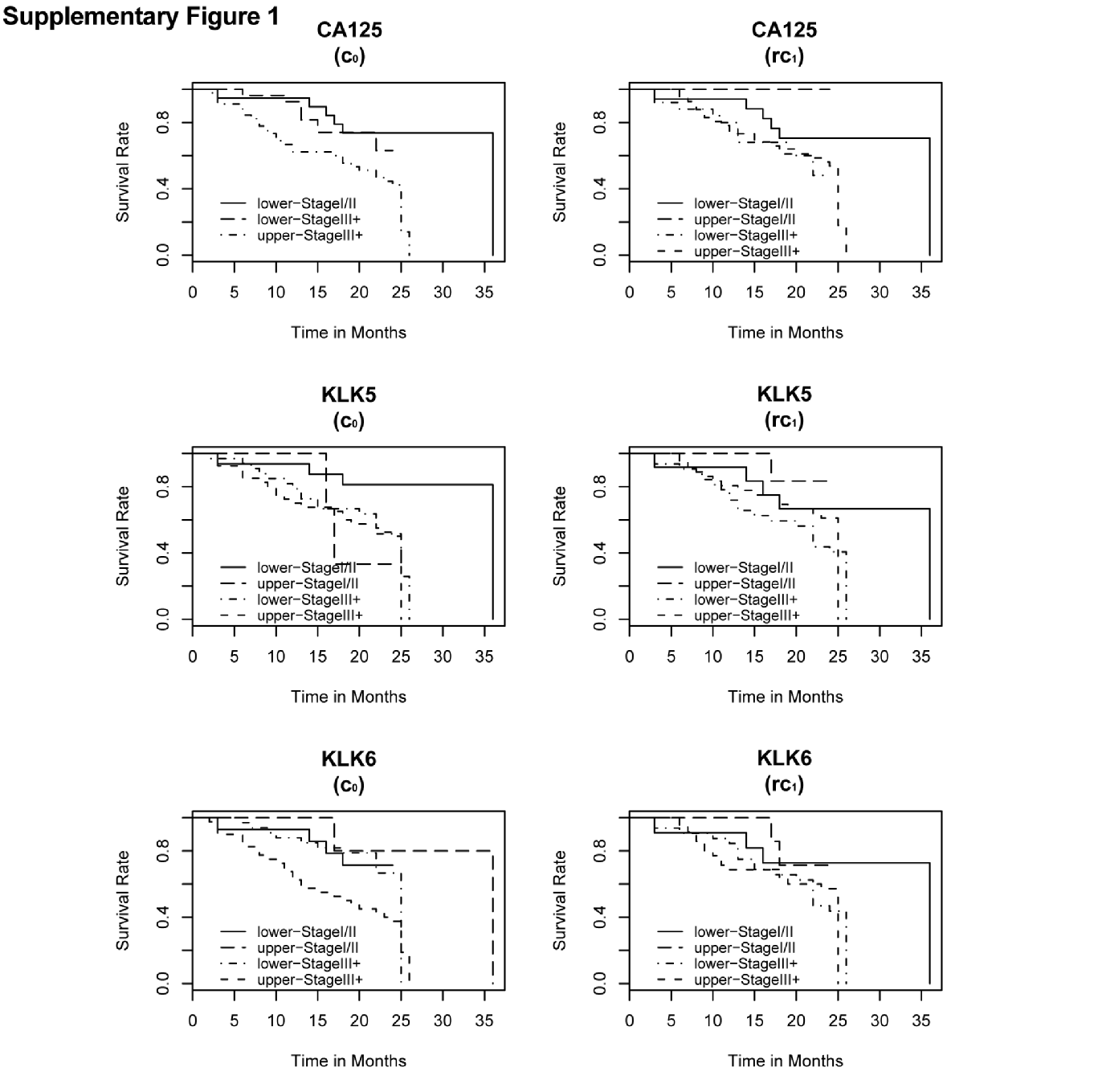


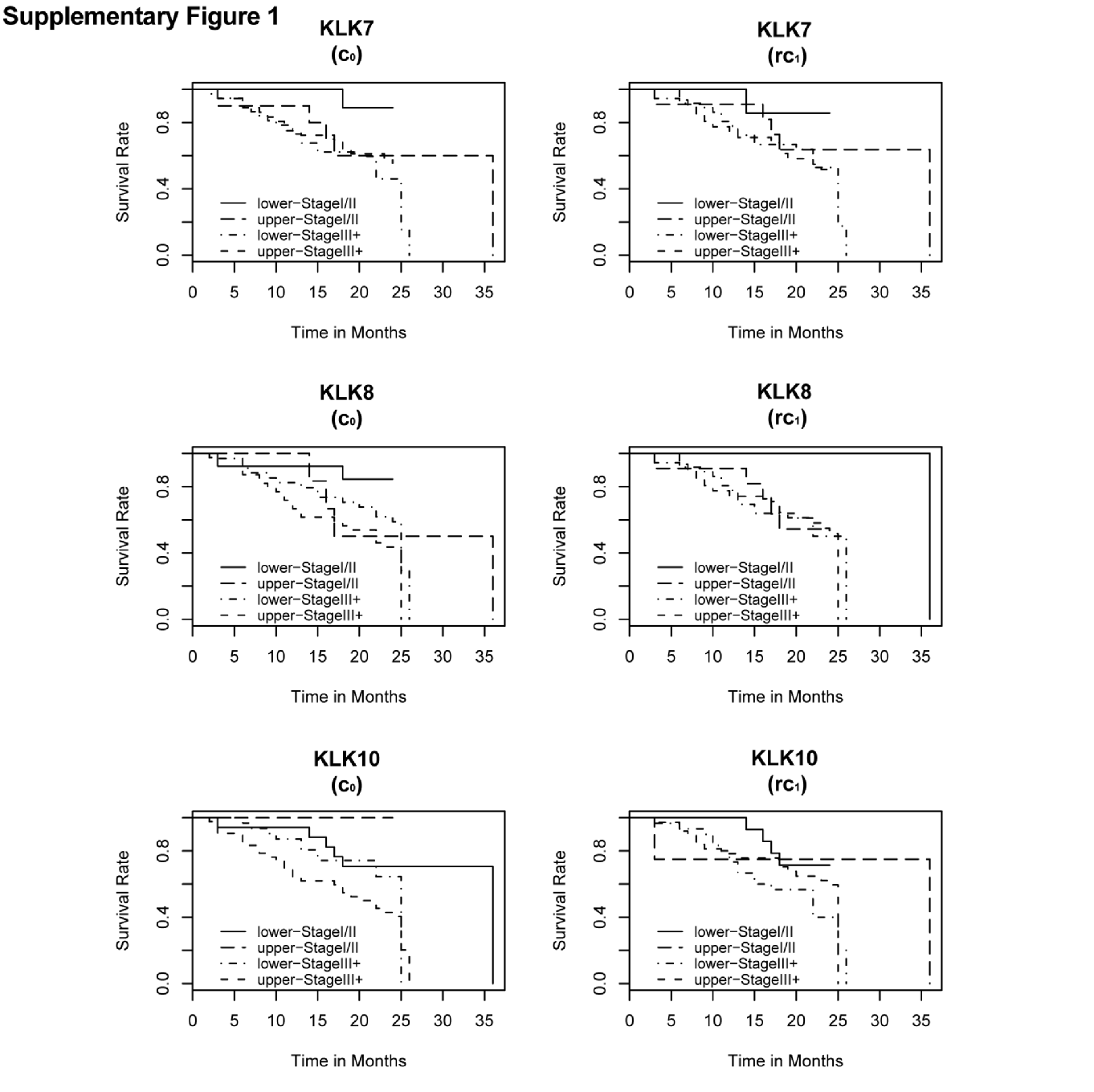


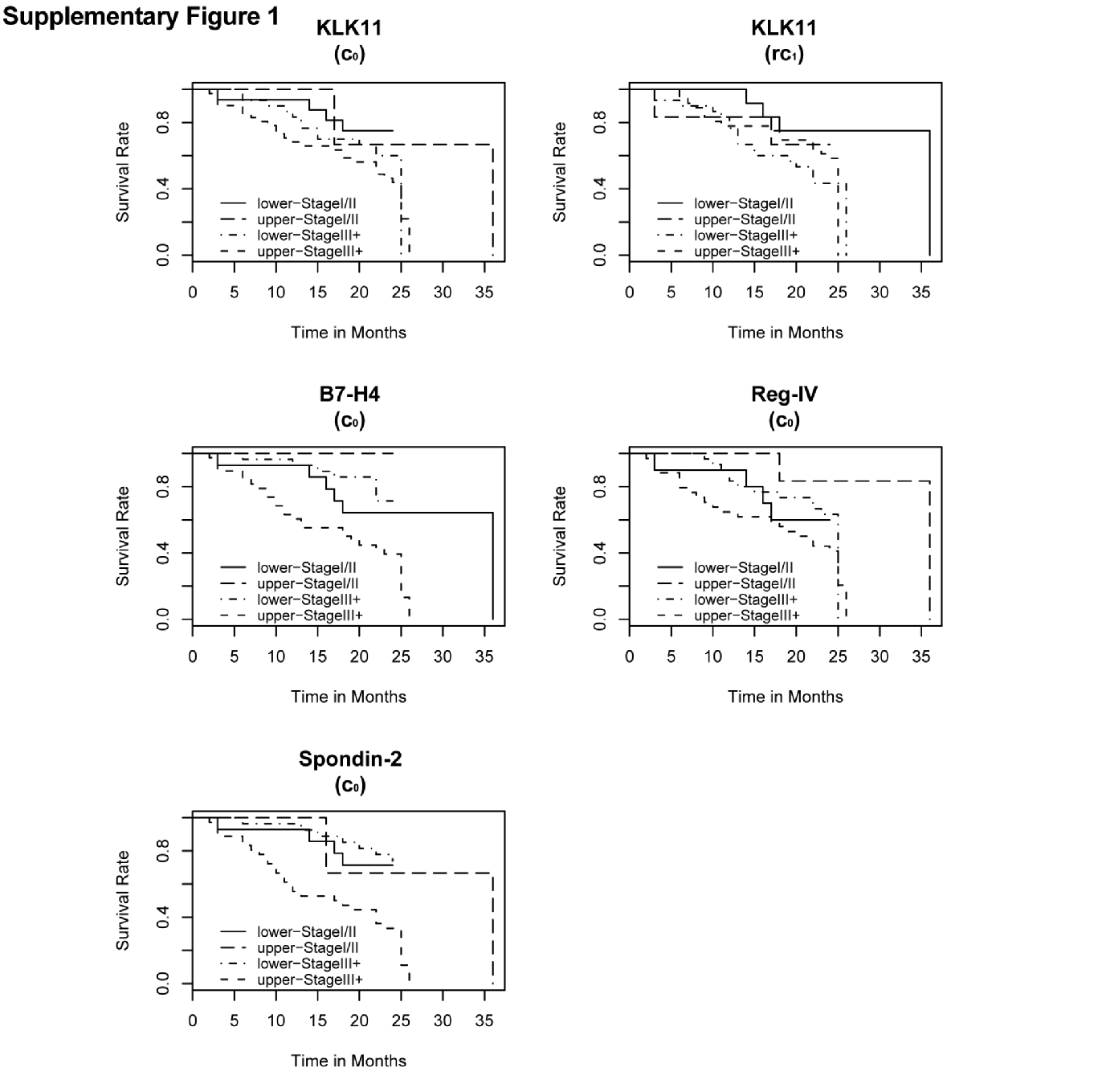


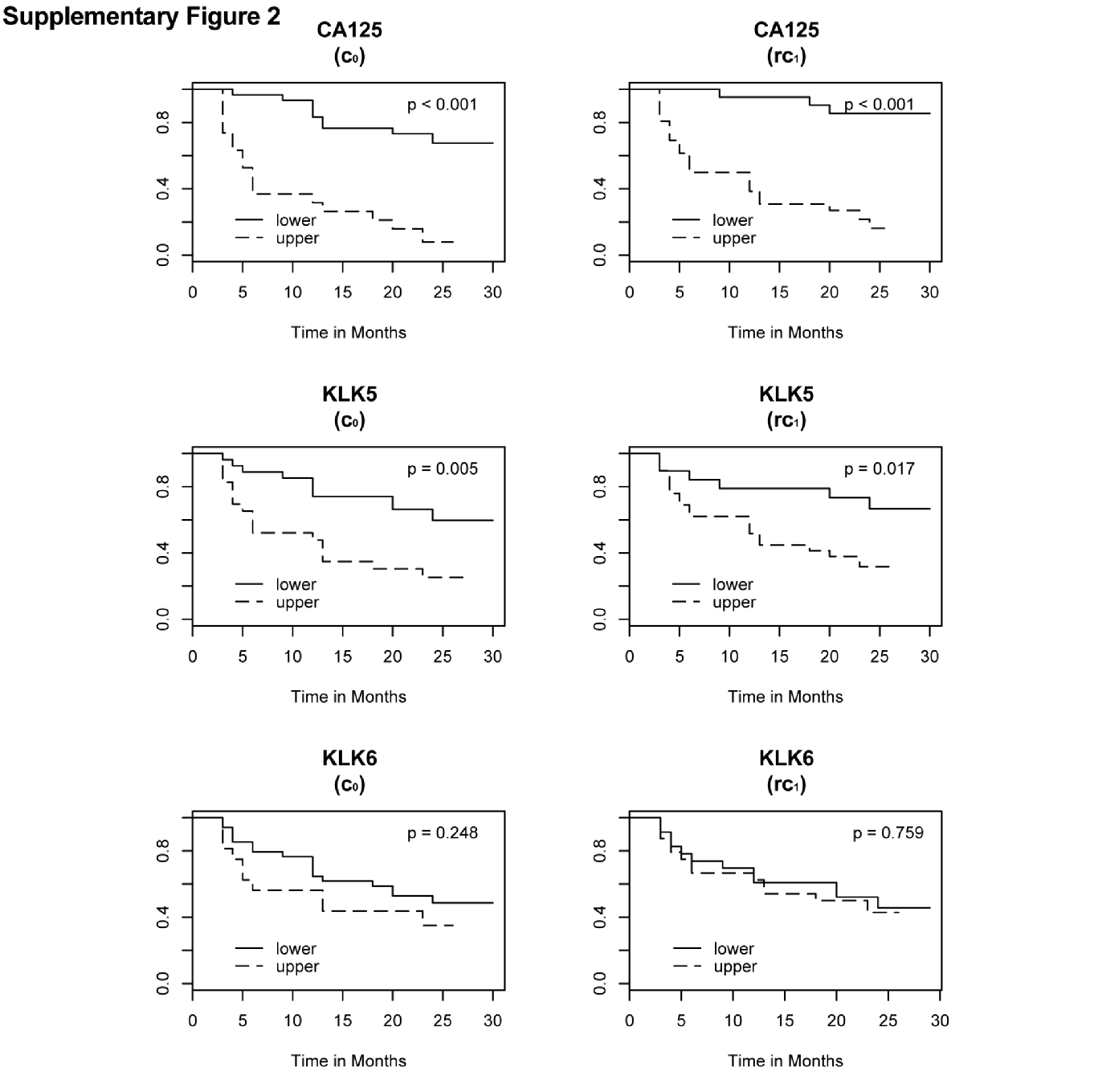


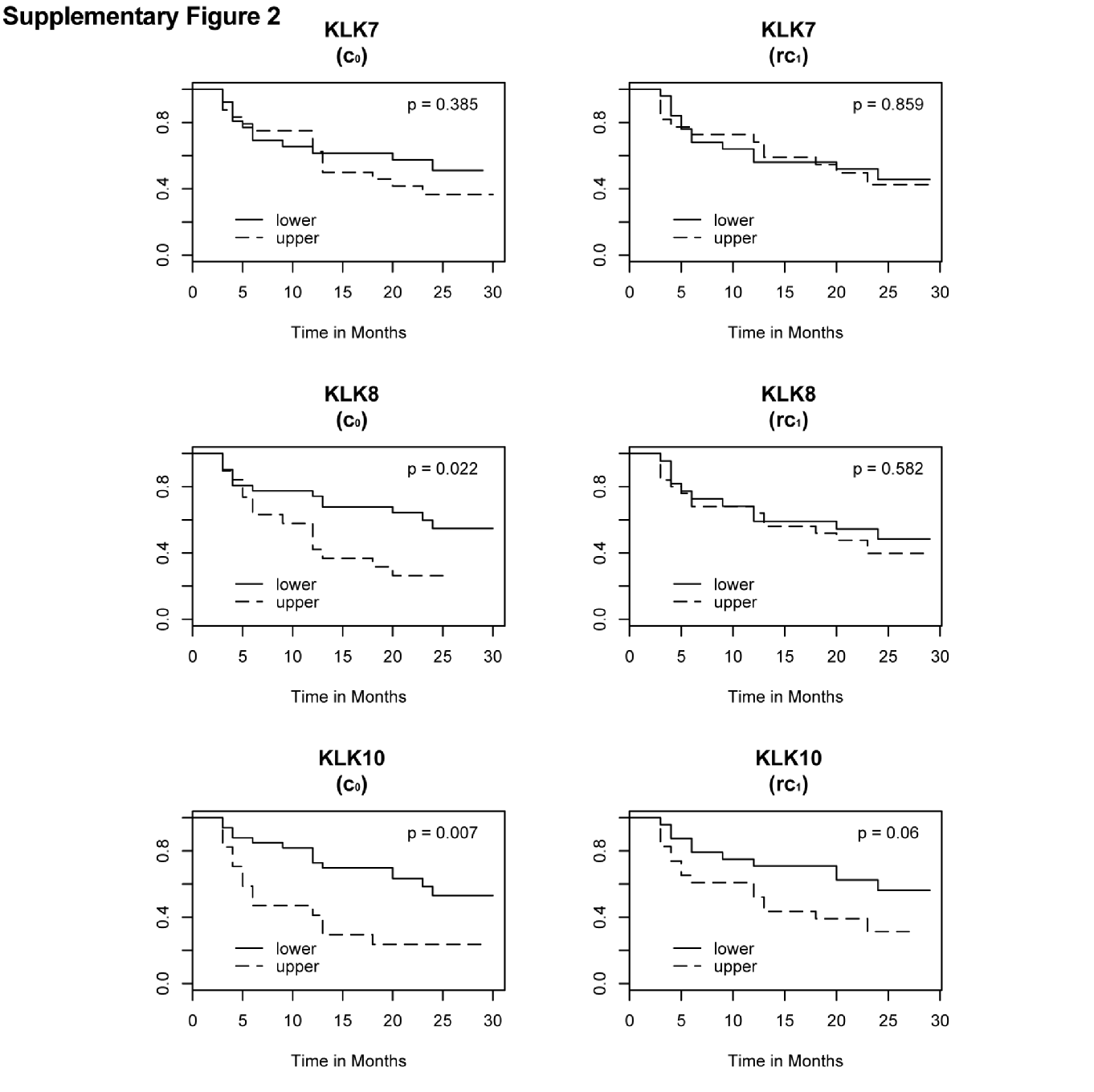


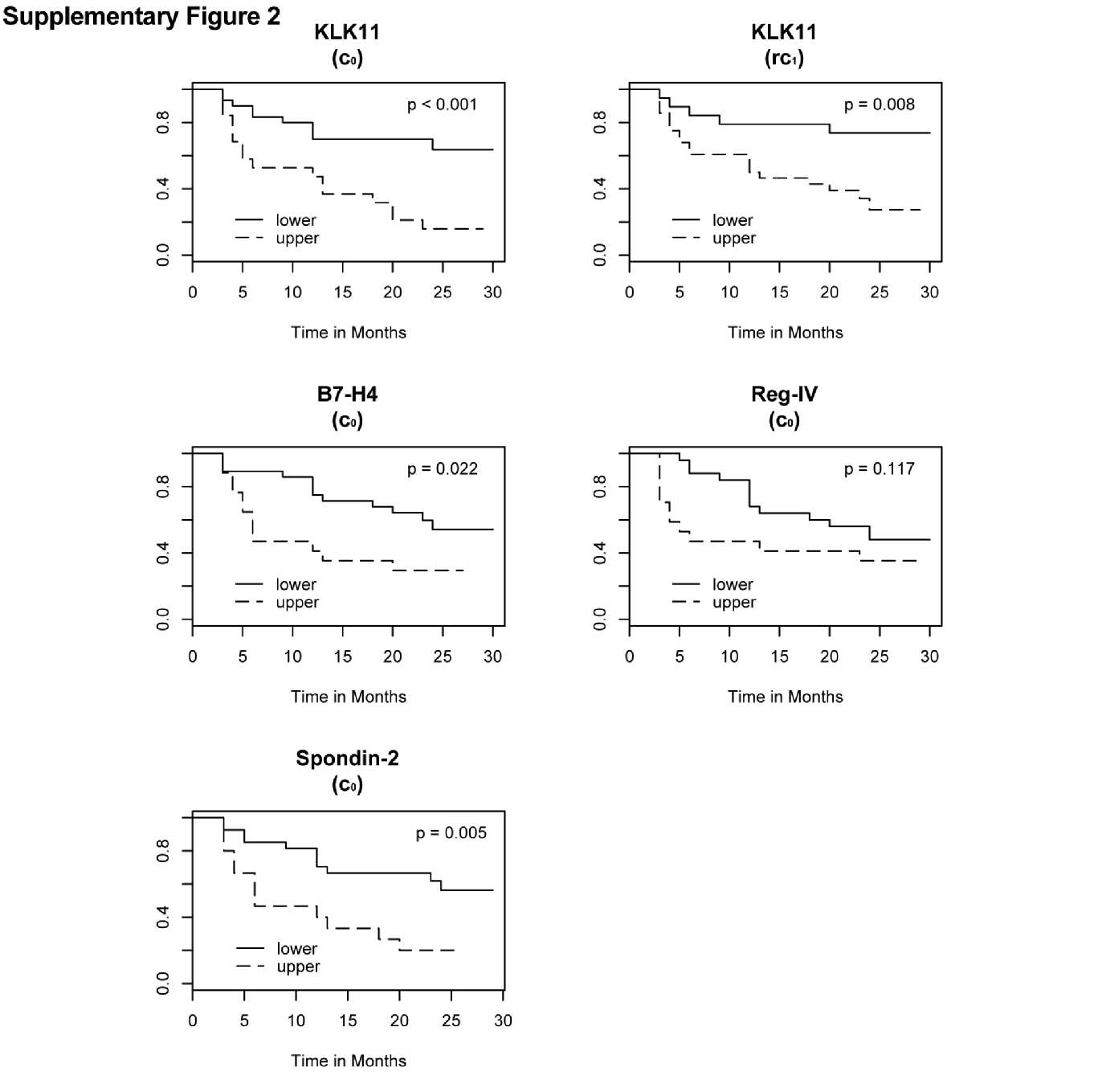

Supplement: Supplementary Information [file 6604630x1.doc]
